# Component tree analysis of cystovirus φ6 nucleocapsid Cryo-EM single particle reconstructions

**DOI:** 10.1371/journal.pone.0188858

**Published:** 2018-01-04

**Authors:** Lucas M. Oliveira, Ze Ye, Al Katz, Alexandra Alimova, Hui Wei, Gabor T. Herman, Paul Gottlieb

**Affiliations:** 1 Department of Computer Science, Graduate Center of the City University of New York, New York, NY, United States of America; 2 Physics Department, City College of New York, New York, NY, United States of America; 3 City University of New York School of Medicine, City College of New York, New York, NY, United States of America; ContraFect Corp, UNITED STATES

## Abstract

The 3-dimensional structure of the nucleocapsid (NC) of bacteriophage φ6 is described utilizing component tree analysis, a topological and geometric image descriptor. The component trees are derived from density maps of cryo-electron microscopy single particle reconstructions. Analysis determines position and occupancy of structure elements responsible for RNA packaging and transcription. Occupancy of the hexameric nucleotide triphosphorylase (P4) and RNA polymerase (P2) are found to be essentially complete in the NC. The P8 protein lattice likely fixes P4 and P2 in place during maturation. We propose that the viral procapsid (PC) is a dynamic structural intermediate where the P4 and P2 can attach and detach until held in place in mature NCs. During packaging, the PC expands to accommodate the RNA, and P2 translates from its original site near the inner 3-fold axis (20 sites) to the inner 5-fold axis (12 sites) with excess P2 positioned inside the central region of the NC.

## Introduction

Bacteriophage φ6 and its relatives are model systems for virus assembly, genome packaging and dsRNA polymerization. The RNA packaging, replication, transcription mechanism, and overall structure resembles that of reoviruses making the species an excellent model system to study these important pathogens. Of particular interest is the molecular, spatial relationships and overall organization of the RNA packaging and transcription elements found in the procapsid (PC) and mature nucleocapsid (NC). We compare the relative location and occupancy of viral portal proteins in the NC and make comparisons to existing models of the pre-packaged PC. A component tree analysis is employed on single particle reconstructions of the NC to identify and locate the viral elements and to estimate protein occupancy.

The φ6 cystovirus species consist of multilayered particles that assemble in a specific order [1, 2]. The initial step in ϕ6 replication is the assembly of an unexpanded procapsid (PC), the structure responsible for the viral messenger RNA (mRNA) packaging, replication to dsRNA, and the early phase transcription of the mRNA. The PC is composed of four proteins–P1, P2, P4, and P7 [1]. It is built from 120 identical P1 proteins organized into a (triangulation number) T = 1 shell containing 60 non-symmetric dimers where each dimer is composed of subunits A and B of the same 3-dimensional fold [3, 4]. The P1 crystal structure has been determined for viruses ϕ6 and ϕ8 and is a trapezoidal shape that accommodates the significant conformational changes that occur during RNA packaging [3].

Ultimately all three segments are replicated to dsRNA segments that are enclosed into a nucleocapsid (NC), the outer layer of which is a shell composed of a matrix assembled of protein P8 [5–7]. The P8 shell is composed of 200 trimers arranged as a T = 13 lattice that partially covers the filled PC [5, 8, 9]. Recently Sun et al. has shown that Ca^+^ can induce transition of the P8 trimer from a closed to open conformation during the outer shell assembly [10]. Two states of the P8 trimers were noted as open and closed allowing domain swapping.

Of particular interest within the cystovirus field has been the location and precise occupancy number of the protein components that constitute the RNA replicative apparatus. This structure consists of the RNA-directed RNA polymerase (RdRP) P2, the hexameric nucleotide triphosphorylase (NTPase) P4, and the packaging factor P7. The initial position of P2 is at the inner 3-fold axis (20 sites) [11] however, after RNA packaging, P2 translates to the inner 5-fold axis (12 sites) as noted in the related species ϕ12 [12]. The position and function of the P7 packaging factor remains controversial. [13] suggested, based on Cryo-EM studies of the PC, that the P7 density overlaps the P2 density implying mutual exclusion of each protein at an inner 3-fold axis site. However difference maps generated between PC reconstructions and mutants lacking either P2 or P7 strongly suggest that P7 stabilizes P2 at the inner three-fold axis prior to RNA packaging [14]. This implies P2 and P7 can interact and that they are located near each other in the vicinity of the inner 3-fold axis. Single particle reconstructions with imposed symmetry of the PC and NC [5, 9] show the protein locations. However, the imposed symmetry forces the non-symmetric RNA into a concentric appearance in the reconstruction. That the hexameric P4 appears pentameric in reconstructions with imposed icosahedral symmetry further demonstrates the limits of a symmetric reconstruction [15, 16].

The structure of the pre-packaged PC has been investigated extensively from single particle reconstructions generated from cryo-EM projections [9, 11, 14, 16–18]. The relative locations of ϕ6 PC proteins have been determined by comparing 3-D reconstructions of the PC to reconstructions of PC mutants lacking P2, P7 or both [14]. The P7 protein was seen to stabilize the RdRP protein P2 at the inner three fold axis prior to RNA packaging [14]. Sen, Heymann, et al. [11] observed ring-like densities on the inner 3-fold axis and estimated that there were approximately 10 copies of P2 per PC particle. In our initial studies single particle reconstructions of cryo-EM images of the NC from the related cystovirus ϕ12 the position of the RdRP was suggested to be at the inner 5 fold axis after RNA packaging and replication [12]. The assumption is that during packaging the RdRP is released from the P7 protein and migrates to the inner 5 fold axis—a position that could facilitate viral RNA transcription.

While there are many sophisticated tools for generating single particle reconstructions of viruses, once a reconstruction is generated, determination of viral elements first rely on visible examination of 2D slices or isosurfaces to isolate densities corresponding to viral elements. An interactive visual methodology that explores spatial relationships and EM densities of the full three-dimensional map will facilitate identification of multiple elements including relative proximity. Therefore component tree analysis can be an effective tool for identification of viral elements when applied to 3D reconstructions.

A component tree is an image descriptor which manifests structural relationships among different parts of an image and have been used in a variety of image processing algorithms, simplification, and object identification [19–22]. A component tree captures the essential structural information about a biological specimen in a way that is independent of the resolution of its density map and the process (e.g. cryo-EM or X-ray crystallography) used to obtain the map. In this study we analyze single particle image reconstruction of the φ6 NC using component tree analysis to determine the RNA replication complex, and protein occupancy and position. Using the spatial relationships found in the NC, we propose that the RdRP, P2, is located in two discreet positions after RNA packaging and replication.

## Materials and methods

### Purification of φ6

The media used to grow the φ6 host cell, *Pseudomonas syringae* LM2333 (provided by the Public Health Research Institute, Newark, NJ) was Luria-Bertani (LB) supplemented with 100 μg/ml ampicillin to inhibit contamination. Plate lysates of ϕ6 were prepared by plating phage dilutions into soft agar with a culture of LM2333. The plates were incubated overnight at room temperature prior to collecting the phage-containing top agar. The cell debris and agar were removed by centrifugation in a Sorvall GSA rotor at 15,000 rpm (3.0 × 10^4^ g) for 30 min at 4°C.

φ6 was collected by centrifugation in a Sorvall T-1270 rotor at 33,000 rpm (1.0 × 10^5^ g) for 2 h at 4°C. The suspended virus pellets were then layered on a 10% to 30% sucrose gradient. Sedimentation centrifugation was at 23,000 rpm (9.4 × 10^4^ g) for 1 h at 15°C using a Beckman SW 40 Ti rotor, after which the virus particle band was visualized by light scattering and the band collected by needle puncture, pelleted by centrifugation (Sorvall T-1270 rotor) at 33,000 rpm (1.0 × 10^5^ g) for 2 h at 4°C, and resuspended in 1 ml of buffer A. Final purification of the phage was by equilibrium centrifugation through 40 to 60% sucrose gradient in buffer A (10 mM KH_2_PO_4_, 1 mM MgSO_4_) using a Beckman SW 40Ti rotor at 23,000 rpm (9.4 × 10^4^ g) overnight at 4°C. The next day the phage band was again visualized by light scattering and collected by tube puncture. The phage sample was then centrifuged with a Sorvall T-1270 rotor at 33,000 rpm (1.0 × 10^5^ g) for 2 h at 4°C and the collected phage particles suspended in 100 μl buffer ACN (10 mM KH_2_PO_4_, 1 mM MgSO_4_, 200 mM NaCl, 0.5 mM CaCl_2_).

### Preparation of φ6 nucleocapsids

The NC particles were isolated by removing the φ6 envelope with Triton X-100 [23, 24]. In summary, the purified φ6 was mixed with 10% Triton X-100 solution in buffer ACN. The NCs were centrifuged at 33,000 rpm (7.5 × 10^4^ g) for 1.5 h at 4°C. Pellets were washed in buffer ACN and then re-suspended in ACN. Although NC concentrations cannot be verified by plaque formation, Triton X-100 envelope removal is essential complete and thus the NC concentrations are approximately equal to the initial whole virion concentrations. Prior analysis of TEM micrographs of NC particles using this procedure has demonstrated nearly complete removal of the viral envelope [25].

### Cryo-electron microscopy and reconstruction

Samples for electron microscopy were plunge frozen in liquid ethane on copper grids with holey carbon film, and imaged with a JEOL 3200 microscope (JEOL Inc. Peabody, MA) operating at 300 kV. Micrographs were acquired at a magnification of 80K on a 4k × 4k CCD detector, a pixel size corresponding to 1.41 Å/pixel. In the reconstruction, a voxel corresponds to a volume of 2.8 × 10^−3^ nm^3^. The defocus range was -1 to -3 μm.

**Fig 1:**
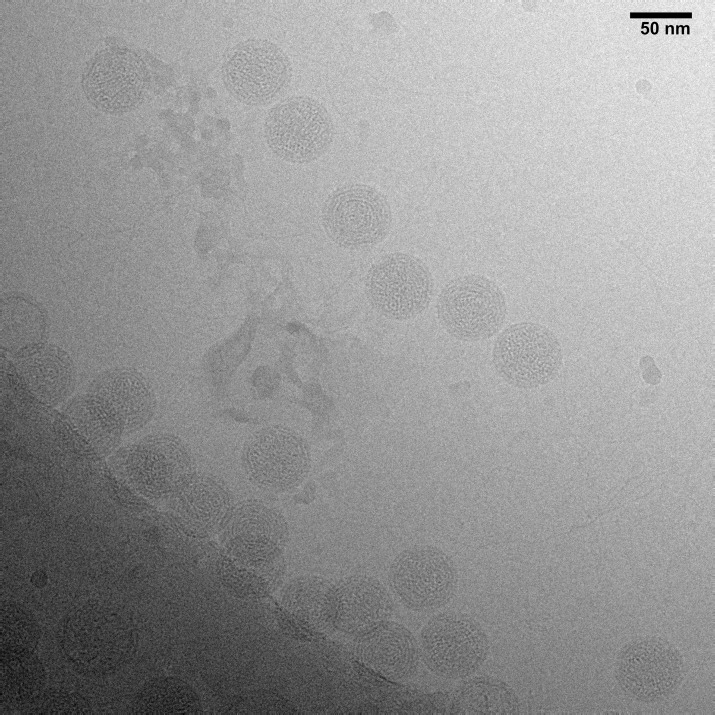
Typical TEM micrograph showing ϕ6 NCs to be selected for boxing.

The NC symmetric single particle reconstruction was computed from a data set consisting of 3450 isolated particles picked from 300 micrographs using XMIPP [26]. A typical TEM micrograph with 11 boxable virions is displayed in Fig 1. Averaged power spectra for the micrographs was generated in SPIDER [27]. The contrast transfer function (CTF) parameters were calculated with CTFFIND3 [28]. Boxed particles were preprocessed for normalization and linear gradient using the RobEM function in the AUTO3DEM software package [29]. The single particle reconstruction was computed in AUTO3DEM using 10 iterations. Initial models were created using the random model method and refined with AUTO3DEM.

### Estimation of protein and RNA segment volumes

Protein volumes were calculated using an average protein density of 1.35 g/cm^3^ (8.12 × 10^2^ Da/nm^3^) [30]. Volumes of the 3 dsRNA segments were estimated based on an average molecular weight of 680 Da/bp and a density of 2.0 g/cm^3^. The RNA volumes are: S segment, 2948 bp, 1.8 × 10^3^ nm^3^; M segment, 4063 bp, 2.3 × 10^3^ nm^3^; and L segment, 6374 bp, 3.6 × 10^3^ nm^3^. The calculated volumes of viral elements were compared to volume estimates determined from counting voxels in each element. At the microscope magnification used, each voxel occupies a volume of 2.8 × 10^−3^ nm^3^. In any real (i.e. noisy) cryo-EM reconstruction, volume estimates are sensitive to the choice of cutoff threshold—a higher threshold gives a smaller volume estimate but enhances resolution of adjacent features. However, volume estimates can still be employed to support assignment of the object to the viral proteins and RNA segments.

### Computation and interpretation of component trees

The component tree was generated and simplified from an NC reconstruction [19]. The component tree was simplified using input parameters λ and κ which only highlighted relevant biological elements and removed noise. The protein volumes estimated from molecular weights served as a guide for the selection of κ values. A larger κ value will combine smaller, adjacent branches into a single element and remove branches corresponding to noise. However, viral subfeatures may also be combined into a single element, e.g. presenting an entire P4 hexamer as a single branch.

Important nodes of the simplified component tree, representing subsets of voxels, were identified. The voxels subsets were exported and displayed with Chimera [31] for the generation of isosurfaces corresponding to the NC viral elements represented by the different component tree nodes.

The interpretation and use of component trees in the exploration of biological structures was presented in detail in [19]. Here we give just a simple graphical illustration based on the reconstruction of the φ6 nucleocapsid (NC).

**Fig 2:**
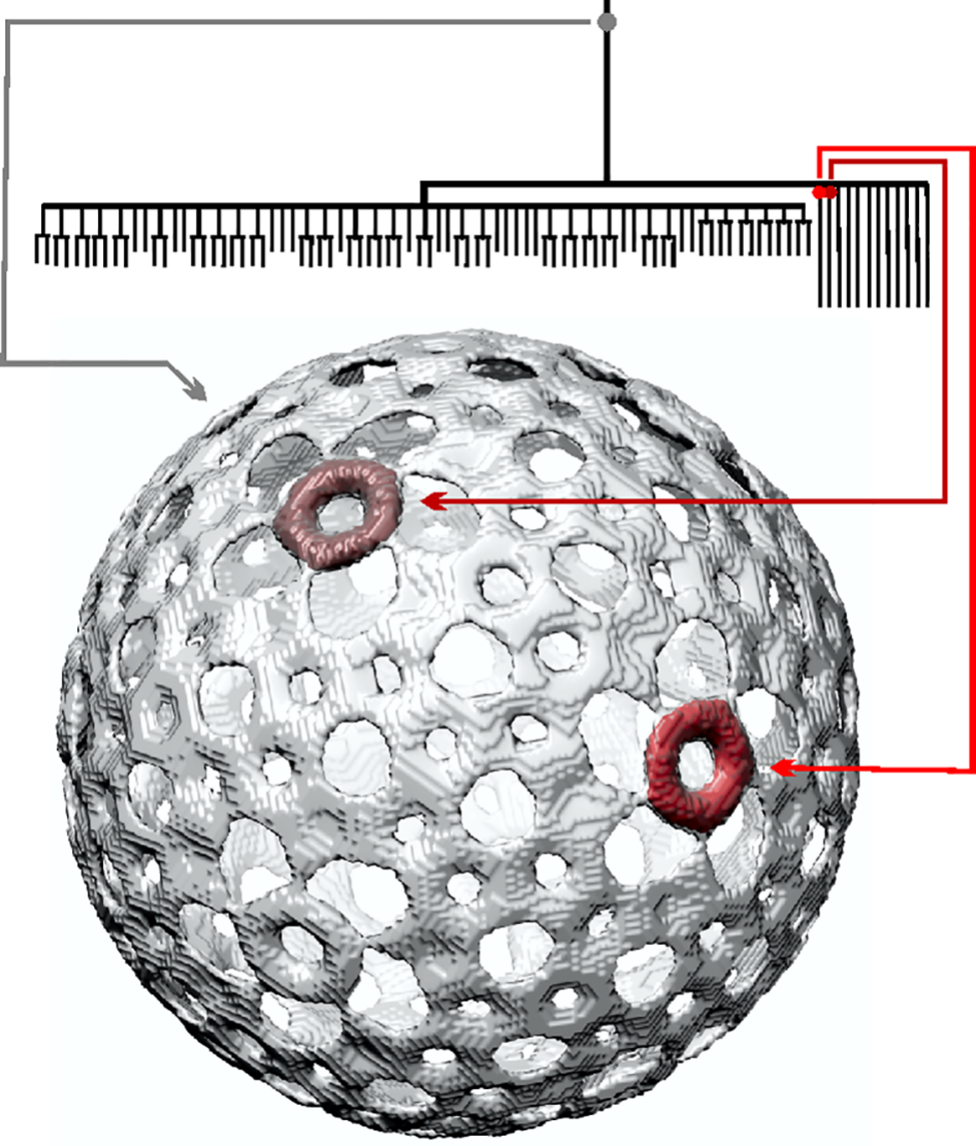
Illustration of the use of a component tree for structural analysis using part of NC component tree to show relationship between nodes of the tree and image voxels.

On the top of Fig 2, is a part of the component tree obtained from the reconstructed NC. Every voxel in the reconstruction is assigned a numerical value (referred to as the “density”) that is the estimated value of the electron density inside the voxel. Every point in the component tree (such points are referred to as “nodes”) determines a set of voxels in the voxel array of the reconstruction. The vertical distance from the top of the tree (called the level) of a node is indicative of a density, all voxels in the set determined by the node will have densities greater than or equal to its level. For example, the large set of voxels that correspond to the node indicated by the small gray disk near the top of Fig 2 has a surface that is displayed in (transparent) gray below the tree. On the other hand, the sets of voxels that correspond to the two nodes further down in the tree indicated by small red disks (of different shades) have surfaces displayed in the corresponding shades of red in the isosurface of Fig 2.

An essential property of the component trees is that if a node B is a “descendant” of another node A, then the set of voxels associated with B is a subset of the voxels associated with A. This is exemplified in Fig 2 by either of the red nodes as B and the gray node as A. The reason why the surface associated with the gray node is displayed as transparent is that we desire to see through it and so identify inside it the locations of the surfaces associated with the red nodes. On the other hand, neither of the red nodes is a “descendant” of the other, in the theory of component trees this implies that the two associated sets of voxels have no elements in common (are disconnected), as can be seen in the surface display part of Fig 2.

The two sets of voxels referred to in the previous paragraph are sufficiently far from each other to make their analysis resistant to the kind of inaccuracies that we are likely to experience in practice: it is clear that there are two of them and, by selecting the levels that are used for their exact definition appropriately, the volumes of the underlying biological entities can be reliably estimated. This is no longer the case if multiple objects (proteins, in our case) are near to each other. This is because the reconstructions contain blurred out versions of the objects and, in order to be able to count the number of them accurately, we need to select a relatively high level. This concept can be illustrated with a simple 1D example described below.

**Fig 3:**
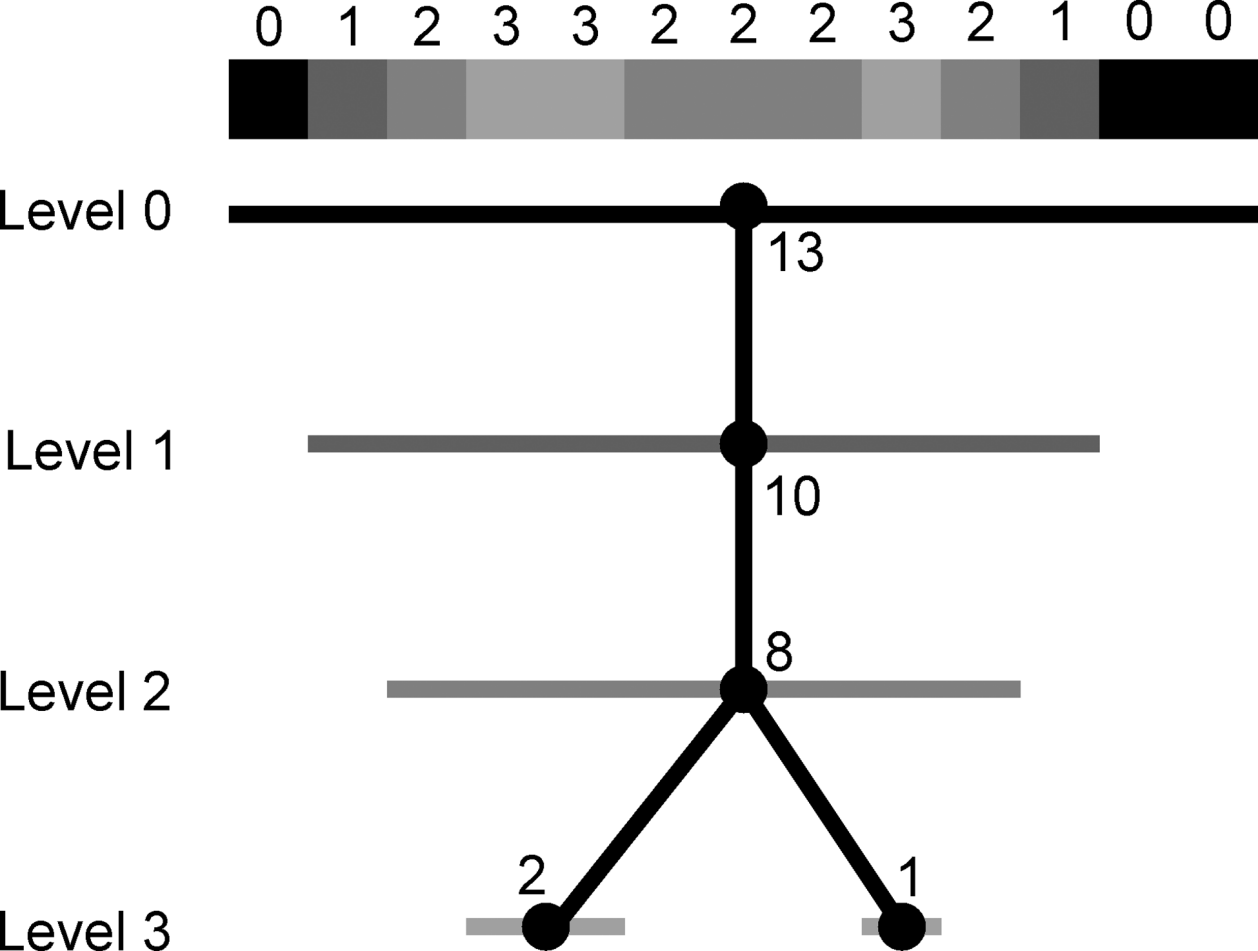
Component tree of a 1D example. The nodes are indicated by black disks. The horizontal bars through the disks identify voxels that belong to the sets of voxels associated with the corresponding nodes and the numbers next to the disks are the numbers of voxels in those sets.

Let us suppose that an imaging process is such that if it is applied to an object with values 0 0 0 0 0 0 3 0 0 0 0 0 0, then the resulting image is 0 0 0 0 0 1 1 1 0 0 0 0 0. This implies (if the imaging process is linear and shift-invariant) that 0 0 0 0 0 3 3 3 0 0 0 0 0 is imaged as 0 0 0 0 1 2 3 2 1 0 0 0 0. Here the actual volume of the object in the original is three and, for threshold 2, the size of the object in the image is also three. However, consider now 0 0 3 3 3 3 0 3 3 3 0 0 0; the actual total volume of the two objects (of different sizes) is seven. The imaging process returns 0 1 2 3 3 2 2 2 3 2 1 0 0. The component tree for this image is shown in Fig 3. There is only one level 2 node since the original two objects blur into each other at that level, however, the size of the node (8 voxels) is close to the combined size of the two objects (7 voxels). We get the correct number of original objects by going to level 3. But at that level the total volume of the two objects in the image is only 3 voxels, which seriously underestimates the actual total volume. This indicates a difficulty that, as we will see, causes a problem in trying to estimate both the number of proteins and the volumes of those proteins in the reconstructed NC. It should be noted that this problem arises from poor resolution of the image collection and processing, not from the generation of the component tree. If the image process returned the exact object, 0 0 3 3 3 3 0 3 3 3 0 0 0, then there would be two level 3 nodes in the tree and the corresponding object would have the correct sizes of 4 and 3 voxels.

## Results and discussion

### NC reconstruction

**Fig 4:**
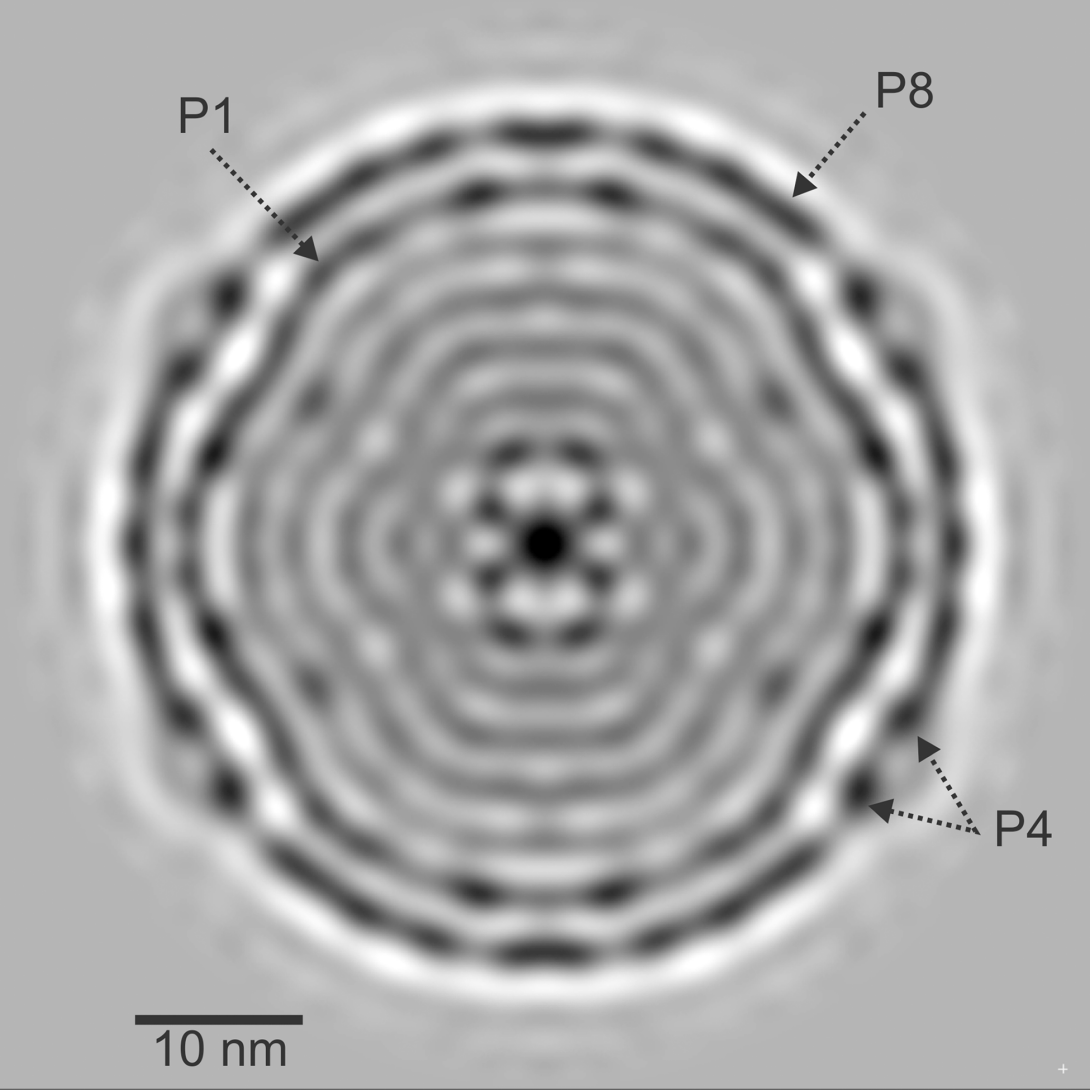
Near central slice of the NC reconstruction showing the NC elements.

**Fig 5:**
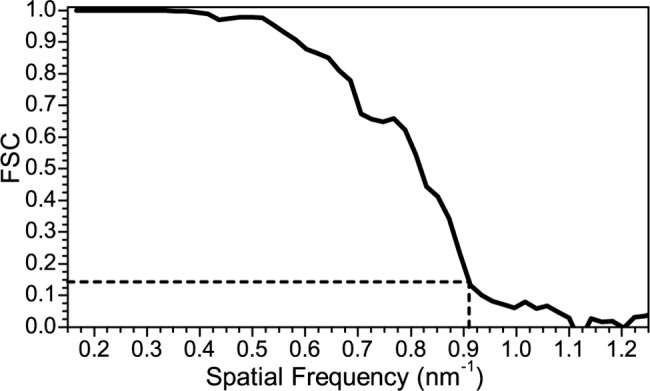
NC Fourier Shell Correlation. Resolution is 1.1 nm using an FSC cutoff of 0.143.

**Fig 6:**
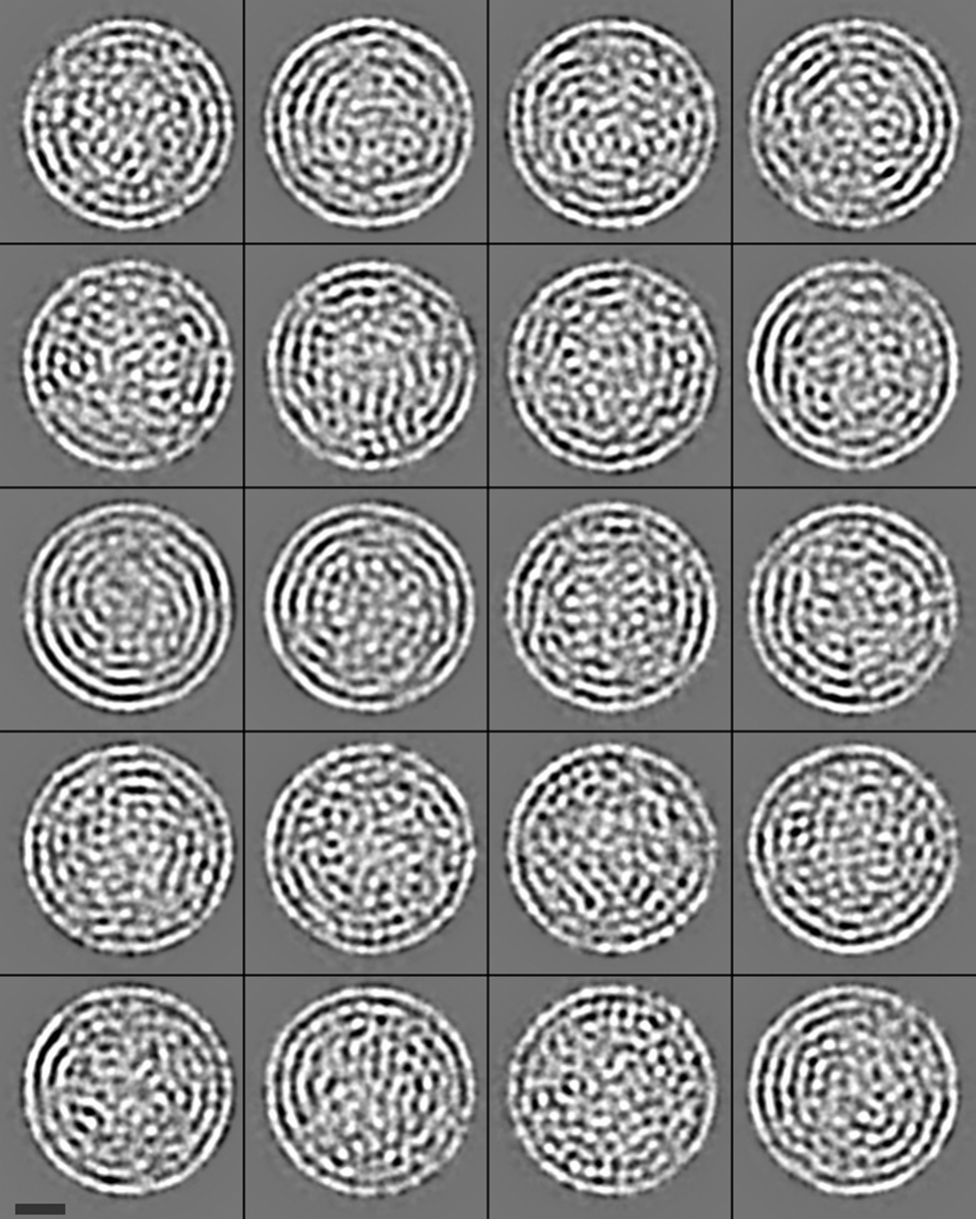
Class Analysis of dsRNA projections without imposed symmetry. The 20 classes have a comparable number of particles in each class indicating a non-symmetric reconstruction could not properly present the RNA. Bar is 10 nm.

Fig 4 shows a central slice of the NC single particle reconstruction with icosahedral symmetry imposed. The resolution of the NC reconstruction is 1.1 nm as determined by Fourier Shell Correlation (FSC) (see Fig 5) using a 0.143 cut-off criterion [32]. The resolution is sufficient to resolve the NC proteins and RNA segments. The slice shows a series of layered structures. The outermost layer is the icosahedral P8 lattice interrupted by the hexameric P4 NTPases sitting at the 5-fold axes [12] and seen in the cross-section as 2 distinct densities at each axes site. P4 densities are comparable to the P8 density, suggesting near 100% occupancy (discussed below). This is in contrast to P4 densities in φ8 and the φ6 PC [14, 16]. Immediately under the P8 lattice is the P1 matrix. The center of the NC exhibits high density regions of the internal proteins (P2 and P7). Below P1 are a series of layers of lower density which correspond to the RNA segments and non-central internal proteins. Small volume, high density regions are observed in the layer directly beneath P1 at the 5-fold symmetry axes sites. An inner layering is observed in the reconstruction, likely a result of imposing 5-fold symmetry on the asymmetric RNA segments. This ring-like artifact has been observed in previous NC single particle reconstructions of cystovirus (φ6 and φ8) [5, 16]. To confirm that the ring-like appearance of the RNA is a result of imposing symmetry, a class analysis of the boxed projection images was performed with P8 and P1 masked and no imposed symmetry. The mask diameter was 39.6 nm, sufficient to remove the outer proteins. A maximum likelihood alignment 2D classification with XIMPP resulted in 20 classes with approximately equal numbers of particles (154 ± 23 projections/class) indicating that the inner region is not packed with RNA possessing 5-fold symmetry. Thus the layering is a result of imposing symmetry. The fact that no clear classes were observed indicates that the RNA exhibits much less symmetry than the outer protein layers and a non-symmetric reconstruction will display the correct RNA orientation. The class averages are shown in Fig 6. Yet in these class images, the RNA still appears somewhat concentric. Indeed in reoviruses the RNAs and RdRPs have been described as having a pseudosymmetric organization [33] Therefore in both the related viral families cystoviruses and reoviruses a highly symmetric NC structure encloses a lower symmetry dsRNA genome. However we note that the component tree analysis denotes three distinct branches, a result in agreement with the 3 distinct dsRNA segments that comprise all the cystovirus species genomes [34].

### Using component trees to identify proteins in the ϕ6 nucleocapsid

**Fig 7:**
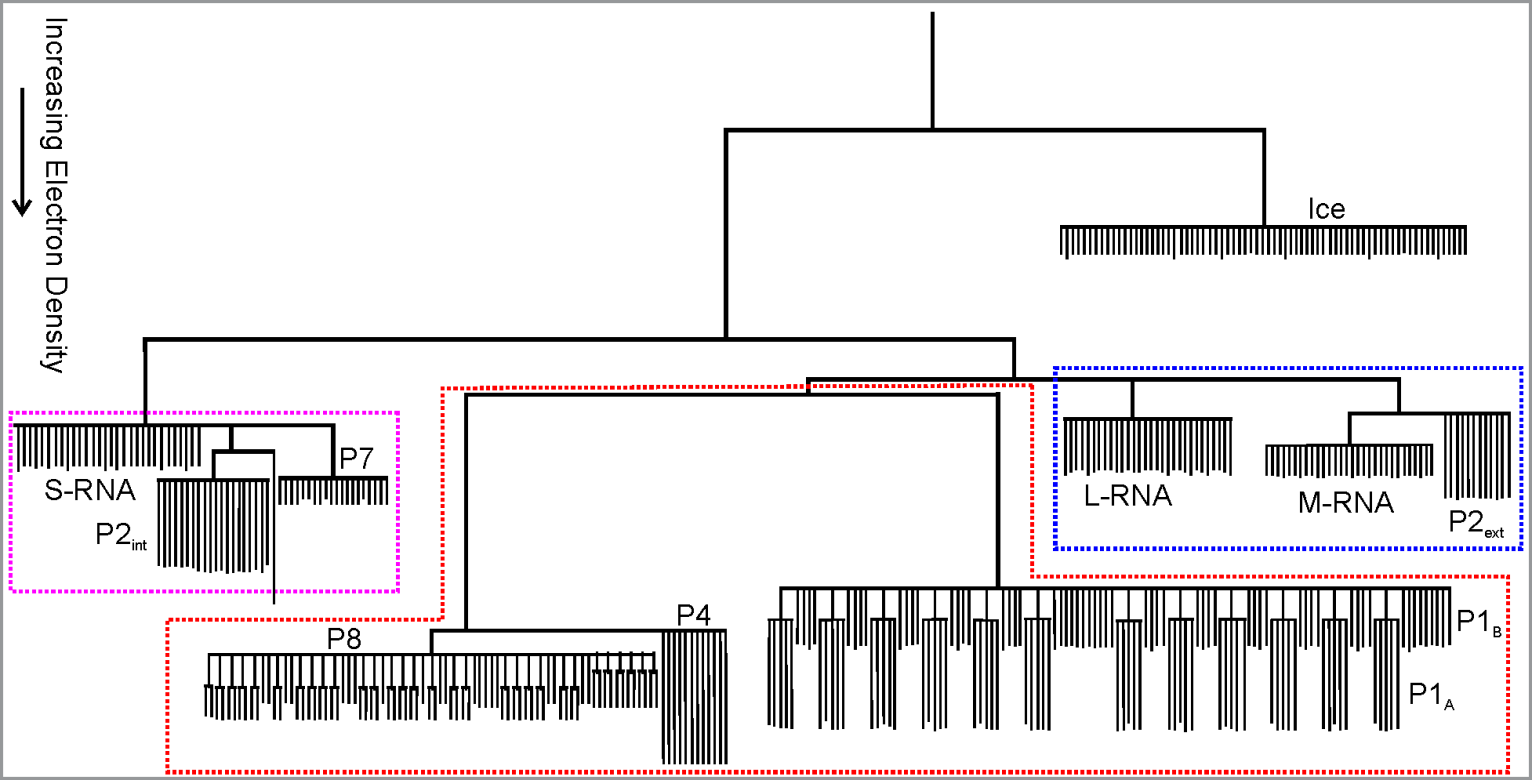
Simplified component tree of the NC reconstruction with branches representing the viral elements identified and labeled in the tree.

The component tree generated from the NC reconstruction was simplified using a κ value such that features less than 2.8 nm^3^ (10^3^ voxels) are considered to be noise and pruned from the tree. λ was chosen to be equal to 1 for the NC tree generation. The simplified NC component tree is plotted in Fig 7. The association of nodes of the component tree to virus components was made by comparing the number and volumes (voxel count) of nodes to the known copy numbers and element sizes, respectively. The low density nodes in the upper right of Fig 7 corresponds to voxels located external to the NC, and thus represent vitrified ice.

**Fig 8:**
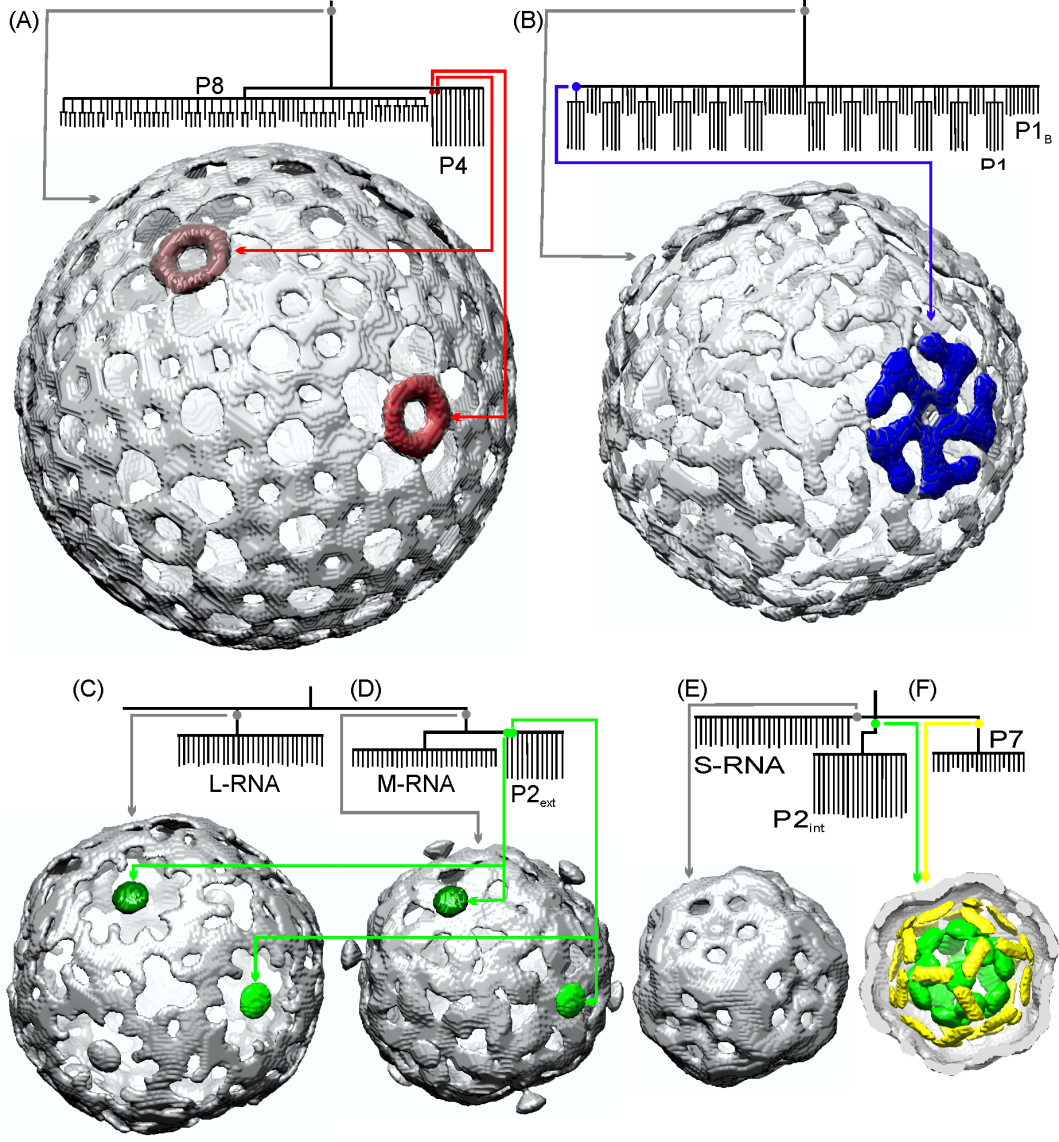
Isosurfaces of viral elements generated from selected voxels in the NC component tree branches identified as (A) P8 and P4; (B) P1; (C) L and (D) M segments of dsRNA and external P2; (E) S segment dsRNA; (F) internal P2 and P7.

At the highest component tree levels (i.e. greatest electron density–region outlined at the bottom of Fig 7 by polygon outlined by a red dotted line) three groups of branches are evident. The group of branches on the left has a volume of 1.2 × 10^4^ nm^3^ and consists of ~75 branches. It is known that the 600 copies of P8 are organized as 200 trimers that form the outer T = 13 lattice [35]. The total volume of the 600 P8 (MW: 16 kDa) is 1.19 × 10^4^ nm^3^, strongly implying that this branch is the P8 lattice. However, in the simplified component tree, the 600 individual copies of P8 are not resolved, likely a result of P8’s relatively small molecular weight and the close proximity of P8 proteins in the tightly-knit matrix. To the right of the P8 branches, there are 12 branches which correspond to the 12 P4 hexamers. The volume of each hexamer branch is 1.6 × 10^2^ nm^3^. This is somewhat smaller than the actual P4 hexamer volume (2.6 × 10^2^ nm^3^), a consequence of choosing a level that is high enough to separate the P4 hexamers from the P8 proteins (see the last two paragraphs of Section 2.5). Employing a smaller κ value (component tree not plotted), each P4 branch demonstrates 5 sub-branches, a consequence of the enforced icosahedral symmetry. An isosurface rendering of the voxels incorporated into this group of branches (voxels corresponding to the node indicated in the Figure by the small gray disk near the top) is shown in Fig 8A. Two of the twelve P4 hexameric packaging motors are shown in red in Fig 8A (voxels corresponding to the red-coded nodes in the Figure). The gray isosurface demonstrates that these branches are indeed located at the outermost layer of the NC, confirming our identification of the branches corresponding to the P8 lattice and the 12 P4 hexamers.

The branches towards the lower right side of the tree (Fig 7) consist of 120 leaves configured as 12 groups of 5 leaves plus 60 additional leaves. It is known [3, 36] that P1 occurs in 2 confirmations termed P1_A_ and P1_B_ with P1_A_ being pentameric with each pentamer surrounded by 5 P1_B_. This reinforces the assignment of these branches to P1 with the 12 groups of 5 leaves corresponding to the 12 P1_A_ pentamers and the other 60 leaves corresponding to the 60 P1_B_ conformations. The volume of the branch is 8.4 × 10^3^ nm^3^, smaller than the total P1 volume (120 P1 proteins with a molecular weight of 85 kDa each, has a total volume of 1.26 × 10^4^ nm^3^). The discrepancy may be a result of some P1 voxels being included in lower density nodes of the tree. An isosurface rendering of P1 is shown in Fig 8B–generated from voxels in the node shown by gray disk in Fig 8B. The elements represented by these branches are located directly beneath the P8 lattice strongly reinforcing our suggestion that the branches represent the P1 matrix. One P1_A_ pentamer generated from the blue-coded node is highlighted, also in blue, in Fig 8B. The location of this branch at the 5-fold axis, directly underneath the P4 packaging motor further demonstrates that it represents the P1_A_ pentamer.

At lower densities than the P8/P1/P4 branches, are two groups of branches. Both groups subdivide into three sets of branches. The branches on the right (designated within the blue rectangle in Fig 7) consist of 2 branches with 30 leaves each and one branch with 12 leaves. The two branches with 30 leaves have volumes of 3.6 × 10^3^ nm^3^ and 2.3 × 10^3^ nm^3^ nearly equal to that of the L and M RNA segment volumes, respectively. In the packaged and expanded PC, P2 is located at the 12 inner 5-fold axes sites. Therefore the 12-leaf branch most likely represents external P2 although the average leaf volume (60 nm^3^) is somewhat less than the volume of a P2 protein (92 nm^3^) due to the phenomenon explained at the end of Section 2.5. Isosurface renderings of these L-RNA and M-RNA branches are shown in Fig 8C and 8D, respectively. The 12 external P2 branches, with two highlighted in green, are also shown in Fig 8C and 8D. That these viral elements are located at the 5-fold axes below the P4 hexamers strongly reinforces our assumption that they are P2. This position below the transcript exit portal would be expected to facilitate the exit of nascent transcripts from the NC. A similar P2 migration from the inner 3-fold to the outer 5-fold axis was proposed for the related φ12 virus [12]; the results reported here strongly support this hypothesis. We again emphasize that the observed structure of the RNA segments is strongly influenced by the symmetry imposed during reconstruction. We do not suggest that the three RNA segments are concentric. However, it is likely that the L segment branch is outermost (immediately below the P1 lattice); the M segment is beneath the L segment; and the S segment is beneath the M segment, following the packaging order of [37].

The three branches on the left side of the tree (designated within the cyan rectangle in Fig 7) consist of one branch with 30 leaves and two branches with 20 leaves each. The volumes of these branches are: 1.6 × 10^3^ nm^3^ (30 leaves, far left in Fig 7); 1.2 × 10^3^ nm^3^ (20 leaves, mid-left); and 9.0 × 10^2^ nm^3^ (20 leaves, towards right side of cyan rectangle). The volume of the S RNA segment is 1.7 × 10^3^ nm^3^. The internal P2 is situated at the 3-fold symmetry axes sites and thus there are potentially 20 copies of internal P2 for a total volume of 1.8 × 10^3^ nm^3^. P7 has a molecular weight of 17.2 kDa and a copy number of 60 [35] and therefore a total volume of 1.25 × 10^3^ nm^3^. Although P7 is dimeric in solution [38], analysis of difference maps of PC reconstructions indicate that 3 P7 densities exist at each of the twenty 3-fold axis sites [13]. Therefore, based on relative volumes and copy number, we suggest the 30 leaf branch is the S-RNA segment; the middle 20 leaf branch is internal P2 and the right 20 leaf branch is P7. An isosurface of the voxels associated with the 30- leaf branch is shown in Fig 8E. This isosurface has a shell-like structure which sits beneath the M segment, consistent with the packaging order, and further reinforcing assignment of this branch to the S-RNA segment. Again, we emphasize that the 30 leaves are an artifact of the imposed symmetry as is the concentric shell-like structures observed in the reconstruction. An isosurface plot of the P2 and P7 branches are shown in Fig 8F with P2 in green and P7 in yellow. It is known that in the prepackaged PC, P7 is closely associated with P2 and initially located near the inner 3-fold axes [13, 14]. The branch locations (internal to the S RNA segment) and their close proximity further supports our assignment of these branches to P2 and P7.

The positions of P2 at both inner and outer sites puts it in proximity to all three dsRNA segments. As indicated above, the outer P2, positioned directly beneath P4, would be available during infection to facilitate the transcription of dsRNA. Similarly, the inner P2 would be available to facilitate transcription of the innermost dsRNA segments, possibly the S and M segments.

**Fig 9:**
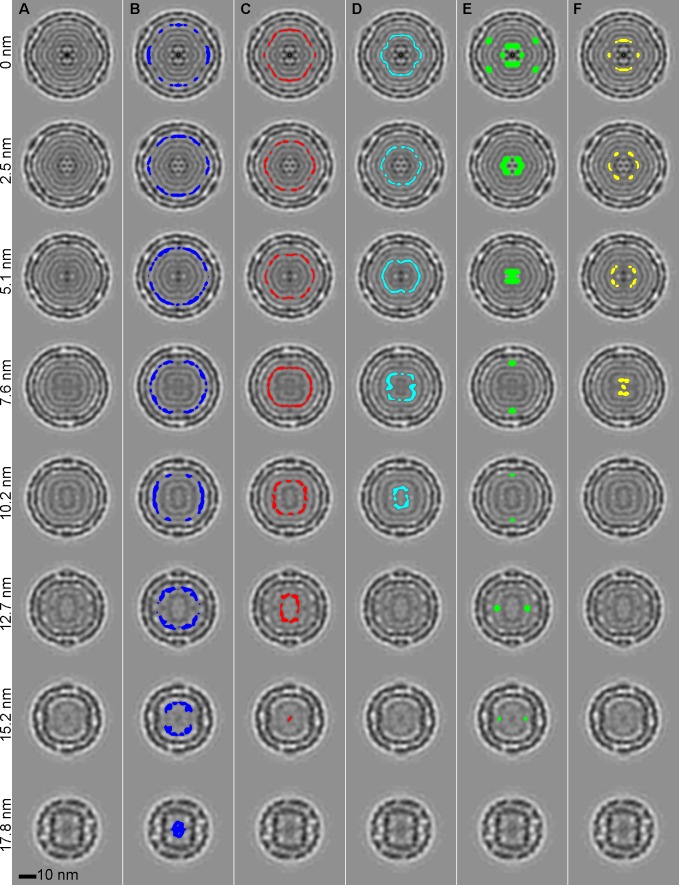
A) Series of NC reconstruction slices with offset distance from central slice indicated at left. Voxels corresponding to component tree branches are highlighted for: B) L-RNA segment (blue); C) M-RNA segment (red); D) S-RNA segment (cyan); E) P2 (green); and F) P7 (yellow).

To further reinforce the assignment of tree branches to the viral elements, the spatial locations of voxels of the six mid-density groups of branches are identified in the reconstruction and highlighted in slices spaced at 2.54 nm intervals in Fig 9. Column A shows non-highlighted slices of the NC. Column B highlights the L segment RNA; column C highlights the M segment RNA; column D highlights the S segment RNA; the inner and outer P2 branches are highlighted in column E; and the P7 branch is highlighted in column F. This panel further identifies that the RNA segments are positioned in the packaging order. The outer P2 are at the 5-fold axes sitting near the L segment. The inner P2 and P7 are centrally located beneath the S segment.

### Protein occupancy in the NC

The occupancy of a protein is estimated as follows. Consider the node in the component tree that identifies the protein in question. As discussed in Section 2.5, such a node determines a set of voxels in the reconstruction and each of these voxels has a (reconstructed) density. Let pd (short for protein density) denote the average of the densities of the voxels in the set. Suppose that we can identify a protein in the NC that we can consider to be 100% occupied (e.g. P1), let its protein density be denoted by PD. Calculate also the average of densities for a set of voxels that are in vitrified ice; let this average be denoted by ID. Then the occupancy of the protein in which we are interested is estimated to be (pd—ID) / (PD—ID).

P1 and P8 are considered to be 100% occupied in the complete NC and their respective nodes are at similar levels in the tree. Since the P4 level (i.e. electron density) is nearly equivalent to that of P1 and P8, P4 in the NC is, therefore, fully occupied. This is significantly greater occupancy than that reported for the unexpanded PC [14, 17], suggesting that the P8 matrix likely stabilizes P4, and that in the absence of the P8 matrix, P4 easily becomes detached from the PC.

Based on electron density, the occupancy of P2 at the external sites is 0.64 for an average of 8 external P2 proteins per NC (0.64 x 12 = 7.68) while the occupancy of P2 at the internal sites is 0.61 for an average of 12 internal P2 per NC (0.61 x 20 = 12.2) for a total of 20 P2 in the NC. Therefore, the PC at assembly (but prior to RNA packaging) has 20 P2, corresponding to 100% occupancy of the unpackaged PC (20 3-fold axes sites). This is significantly higher P2 occupancy than reported for the recombinant PC assembled in *E coli* [14, 17]. The NC that is analyzed in this research is assembled during the entire replication cycle and includes the addition of the P8 lattice. We speculate that the P8 lattice insures that the RNA portal elements are stabilized *in situ* in order to complete the entire viral architecture—a condition not realized in the recombinant PC. Therefore the presence of the P8 lattice maintains the NC at near complete occupancy of P2 and P4, a condition not realized in recombinant PCs. Thus it would be of interest to examine the final P4 and P2 occupancy of the cystovirus species ϕ8 that normally lacks the P8 lattice and determine if it matures with 100% P4 and P2 occupancy levels.

Electron density estimates put the occupancy of P7 at 0.59 which may indicate that in the unexpanded PC, P7 sites may not be fully occupied. However, it is possible that some P7 may have migrated away from the central region during expansion. Since P7 is a relatively small protein, the spatial resolution of the reconstruction may not be sufficient to detect isolated P7.

### Conclusions

The study presented above demonstrates a new tool for the analysis of EM image reconstructions of particles. Most notable is the observation that all the major structural elements of a well classified viral particle are identifiable and located in the reconstruction. Copy number and occupancy are quantified by the analysis. We demonstrate that component tree analysis provides a useful tool for understanding the spatial organization of functional elements of viral reconstructions. Even in the absence of distinct symmetry, component tree analysis may be applicable for pleomorphic viruses as well.

The analysis demonstrates that the P2 and P4 occupancy is greater in the NC than a particle lacking the P8 matrix [14, 17]. The occupancy of the P4 hexamer is near 100%. This observation has been previously reported based upon tomogram and single particle reconstructions of the PC but never rigorously shown in an NC. The low occupancy of the PC P4 hexamer could account for the notion that only one (or at the most two) portals are active during RNA packaging.

A surprising result is the observation that in the NC there are two major P2 classes identified at two distinct locations–the 20 sites near center of the NC in proximity to the S segment; and 12 sites in the outer region between the M and L segments. We refer to the near center sites as internal P2 and the 12 outer sites as external P2. The existence of two distinct P2 locations lends credence to the idea that some P2 migrates from the inner 3-fold axis to the outer 5-fold axis. We further speculate that the two P2 classes guarantee association with the three dsRNA genome segments allowing efficient transcription at initiation of a subsequent infection. The total number of P2 in both classes adds to 20, i.e. 100% occupancy in the unexpanded PC.

## References

[1] Gottlieb P, Metzger S, Romantschuk M, Carton J, Strassman J, Bamford DH, et al Nucleotide sequence of the middle dsRNA segment of bacteriophage phi6: placement of the genes of membrane-associated proteins. Virology. 1988;163(1):183–90. 3347997 10.1016/0042-6822(88)90245-0

[2] Gottlieb P, Strassman J, Qiao XY, Frucht A, Mindich L. In vitro replication, packaging, and transcription of the segmented double-stranded RNA genome of bacteriophage φ6: studies with procapsids assembled from plasmid-encoded proteins. J Bacteriol. 1990;172(10):5774–82. . 2211512 10.1128/jb.172.10.5774-5782.1990 PMC526894

[3] Nemecek D, Boura E, Wu W, Cheng N, Plevka P, Qiao J, et al Subunit Folds and Maturation Pathway of a dsRNA Virus Capsid. Structure. 2013;21(8):1374–83. doi: 10.1016/j.str.2013.06.007 ; PubMed Central PMCID: PMC3742642. 23891288 10.1016/j.str.2013.06.007 PMC3742642

[4] El Omari K, Meier C, Kainov D, Sutton G, Grimes JM, Poranen MM, et al Tracking in atomic detail the functional specializations in viral RecA helicases that occur during evolution. Nucleic Acids Res. 2013 doi: 10.1093/nar/gkt713 ; PubMed Central PMCID: PMC3814363. 23939620 10.1093/nar/gkt713 PMC3814363

[5] Butcher SJ, Dokland T, Ojala PM, Bamford DH, Fuller SD. Intermediates in the assembly pathway of the double-stranded RNA virus φ6. EMBO J. 1997;16(14):4477–87. doi: 10.1093/emboj/16.14.4477 . 9250692 10.1093/emboj/16.14.4477 PMC1170074

[6] Olkkonen VM, Bamford DH. The nucleocapsid of the lipid-containing double-stranded RNA bacteriophage phi 6 contains a protein skeleton consisting of a single polypeptide species. J Virol. 1987;61(8):2362–7. Epub 1987/08/01. ; PubMed Central PMCID: PMC255646. 3599179 10.1128/jvi.61.8.2362-2367.1987 PMC255646

[7] Olkkonen VM, Ojala PM, Bamford DH. Generation of infectious nucleocapsids by *in vitro* assembly of the shell protein on to the polymerase complex of the dsRNA bacteriophage phi 6. J Mol Biol. 1991;218(3):569–81. Epub 1991/04/05. . 2016747 10.1016/0022-2836(91)90702-8

[8] Bamford DH, Mindich L. Electron microscopy of cells infected with nonsense mutants of bacteriophage φ6. Virology. 1980;107(1):222–8. Epub 1980/11/01. . 7445427 10.1016/0042-6822(80)90287-1

[9] Huiskonen JT, de Haas F, Bubeck D, Bamford DH, Fuller SD, Butcher SJ. Structure of the bacteriophage φ6 nucleocapsid suggests a mechanism for sequential RNA packaging. Structure. 2006;14(6):1039–48. doi: 10.1016/j.str.2006.03.018 . 16765897 10.1016/j.str.2006.03.018

[10] Sun Z, El Omari K, Sun X, Ilca SL, Kotecha A, Stuart DI, et al Double-stranded RNA virus outer shell assembly by bona fide domain-swapping. Nat Commun. 2017;8:14814 Epub 2017/03/14. doi: 10.1038/ncomms14814 ; PubMed Central PMCID: PMC5355851. 28287099 10.1038/ncomms14814 PMC5355851

[11] Sen A, Heymann JB, Cheng N, Qiao J, Mindich L, Steven AC. Initial location of the RNA-dependent RNA polymerase in the bacteriophage φ6 procapsid determined by cryo-electron microscopy. J Biol Chem. 2008;283(18):12227–31. Epub 2008/02/22. doi: 10.1074/jbc.M710508200 ; PubMed Central PMCID: PMC2335345. 18287088 10.1074/jbc.M710508200 PMC2335345

[12] Wei H, Cheng RH, Berriman J, Rice WJ, Stokes DL, Katz A, et al Three-dimensional Structure of the Enveloped Bacteriophage ϕ12: An Incomplete T = 13 Lattice is Superposed on an Enclosed T = 1 Shell. PLoS One. 2009;4(9):e6850 PubMed Central PMCID: PMC2733035. doi: 10.1371/journal.pone.0006850 19727406 10.1371/journal.pone.0006850 PMC2733035

[13] Nemecek D, Qiao J, Mindich L, Steven AC, Heymann JB. Packaging accessory protein P7 and polymerase P2 have mutually occluding binding sites inside the bacteriophage φ6 procapsid. J Virol. 2012;86(21):11616–24. doi: 10.1128/JVI.01347-12 ; PubMed Central PMCID: PMC3486324. 22896624 10.1128/JVI.01347-12 PMC3486324

[14] Katz G, Wei H, Alimova A, Katz A, Morgan DG, Gottlieb P. Protein P7 of the Cystovirus ϕ6 is Located at the Three-Fold Axis of the Unexpanded Procapsid. PloS One. 2012;7(10):e47489 doi: 10.1371/journal.pone.0047489. PubMed Central PMCID: PMC3471842. 23077625 10.1371/journal.pone.0047489 PMC3471842

[15] de Haas F, Paatero AO, Mindich L, Bamford DH, Fuller SD. A symmetry mismatch at the site of RNA packaging in the polymerase complex of dsRNA bacteriophage φ6. J Mol Biol. 1999;294(2):357–72. doi: 10.1006/jmbi.1999.3260 . 10610764 10.1006/jmbi.1999.3260

[16] Jaalinoja HT, Huiskonen JT, Butcher SJ. Electron cryomicroscopy comparison of the architectures of the enveloped bacteriophages φ6 and φ8. Structure. 2007;15(2):157–67. doi: 10.1016/j.str.2006.12.004 . 17292834 10.1016/j.str.2006.12.004

[17] Nemecek D, Heymann JB, Qiao J, Mindich L, Steven AC. Cryo-electron tomography of bacteriophage φ6 procapsids shows random occupancy of the binding sites for RNA polymerase and packaging NTPase. J Struct Biol. 2010;171(3):389–96. Epub 2010/06/12. doi: 10.1016/j.jsb.2010.06.005 ; PubMed Central PMCID: PMC2910799. 20538059 10.1016/j.jsb.2010.06.005 PMC2910799

[18] Jiang W, Chiu W. Cryoelectron microscopy of icosahedral virus particles. Methods Mol Biol. 2007;369:345–63. Epub 2007/07/28. doi: 10.1007/978-1-59745-294-6_17 . 17656759 10.1007/978-1-59745-294-6_17

[19] Oliveira L, Kong TY, Herman GT. Using Component Trees to Explore Biological Structures In: Herman GT, Frank J, editors. Computational Methods for Three-Dimensional Microscopy Reconstruction. Applied and Numerical Harmonic Analysis: Springer New York; 2014 p. 221–55.

[20] Herman GT, Kong TY, Oliveira L. Provably Robust Simplification of Component Trees of Multidimensional Images In: Brimkov VE, Barneva RP, editors. Digital Geometry Algorithms. Lecture Notes in Computational Vision and Biomechanics. 2: Springer Netherlands; 2012 p. 27–69.

[21] Herman GT, Kong TY, Oliveira LM. Tree representation of digital picture embeddings. Journal of Visual Communication and Image Representation. 2012;23(6):883–91. doi: 10.1016/j.jvcir.2012.05.007

[22] Sarioz D, Kong TY, Herman GT. History Trees as Descriptors of Macromolecular Structures In: Bebis G, Boyle R, Parvin B, Koracin D, Remagnino P, Nefian A, et al, editors. Advances in Visual Computing. Lecture Notes in Computer Science. 4291: Springer Berlin Heidelberg; 2006 p. 263–72.

[23] Steely HT Jr., Lang D. Electron microscopy of bacteriophage phi 6 nucleocapsid: two-dimensional image analysis. J Virol. 1984;51(2):479–83. ; PubMed Central PMCID: PMC254462. 6205171 10.1128/jvi.51.2.479-483.1984 PMC254462

[24] Gottlieb P, Wei H, Potgieter C, Toporovsky I. Characterization of φ12, a bacteriophage related to φ6: nucleotide sequence of the small and middle double-stranded RNA. Virology. 2002;293(1):118–24. Epub 2002/02/21. doi: 10.1006/viro.2001.1288 . 11853405 10.1006/viro.2001.1288

[25] Block KA, Trusiak A, Katz A, Gottlieb P, Alimova A, Wei H, et al Disassembly of the cystovirus ϕ6 envelope by montmorillonite clay. MicrobiologyOpen. 2014;3(1):42–51. doi: 10.1002/mbo3.148 ; PubMed Central PMCID: PMC3937728. 24357622 10.1002/mbo3.148 PMC3937728

[26] Sorzano CO, Marabini R, Velazquez-Muriel J, Bilbao-Castro JR, Scheres SH, Carazo JM, et al XMIPP: a new generation of an open-source image processing package for electron microscopy. Journal of Structural Biology. 2004;148(2):194–204. Epub 2004/10/13. doi: 10.1016/j.jsb.2004.06.006 . 15477099 10.1016/j.jsb.2004.06.006

[27] Frank J, Radermacher M, Penczek P, Zhu J, Li Y, Ladjadj M, et al SPIDER and WEB: processing and visualization of images in 3D electron microscopy and related fields. Journal of Structural Biology. 1996;116(1):190–9. doi: 10.1006/jsbi.1996.0030 . 8742743 10.1006/jsbi.1996.0030

[28] Mindell JA, Grigorieff N. Accurate determination of local defocus and specimen tilt in electron microscopy. Journal of Structural Biology. 2003;142(3):334–47. Epub 2003/06/05. . 12781660 10.1016/s1047-8477(03)00069-8

[29] Yan X, Sinkovits RS, Baker TS. AUTO3DEM—an automated and high throughput program for image reconstruction of icosahedral particles. Journal of Structural Biology. 2007;157(1):73–82. Epub 2006/10/13. doi: 10.1016/j.jsb.2006.08.007 ; PubMed Central PMCID: PMC1847775. 17029842 10.1016/j.jsb.2006.08.007 PMC1847775

[30] Fischer H, Polikarpov I, Craievich AF. Average protein density is a molecular-weight-dependent function. Protein Sci. 2004;13(10):2825–8. Epub 2004/09/25. doi: 10.1110/ps.04688204 ; PubMed Central PMCID: PMC2286542. 15388866 10.1110/ps.04688204 PMC2286542

[31] Pettersen EF, Goddard TD, Huang CC, Couch GS, Greenblatt DM, Meng EC, et al UCSF Chimera—a visualization system for exploratory research and analysis. J Comput Chem. 2004;25(13):1605–12. Epub 2004/07/21. doi: 10.1002/jcc.20084 . 15264254 10.1002/jcc.20084

[32] Rosenthal PB, Henderson R. Optimal determination of particle orientation, absolute hand, and contrast loss in single-particle electron cryomicroscopy. J Mol Biol. 2003;333(4):721–45. Epub 2003/10/22. S0022283603010222 [pii]. . 14568533 10.1016/j.jmb.2003.07.013

[33] Liu H, Cheng L. Cryo-EM shows the polymerase structures and a nonspooled genome within a dsRNA virus. Science. 2015;349(6254):1347–50. Epub 2015/09/19. doi: 10.1126/science.aaa4938 . 26383954 10.1126/science.aaa4938

[34] Mindich L, Qiao X, Qiao J, Onodera S, Romantschuk M, Hoogstraten D. Isolation of additional bacteriophages with genomes of segmented double-stranded RNA. Journal of Bacteriology. 1999;181(15):4505–8. . 10419946 10.1128/jb.181.15.4505-4508.1999 PMC103579

[35] Poranen MM, Paatero AO, Tuma R, Bamford DH. Self-assembly of a viral molecular machine from purified protein and RNA constituents. Mol Cell. 2001;7(4):845–54. . 11336707 10.1016/s1097-2765(01)00228-3

[36] El Omari K, Sutton G, Ravantti JJ, Zhang H, Walter TS, Grimes JM, et al Plate Tectonics of Virus Shell Assembly and Reorganization in Phage Phi8, a Distant Relative of Mammalian Reoviruses. Structure. 2013;21(8):1384–95. doi: 10.1016/j.str.2013.06.017 ; PubMed Central PMCID: PMC3737474. 23891291 10.1016/j.str.2013.06.017 PMC3737474

[37] Qiao X, Casini G, Qiao J, Mindich L. In vitro packaging of individual genomic segments of bacteriophage phi 6 RNA: serial dependence relationships. Journal of Virology. 1995;69(5):2926–31. Epub 1995/05/01. ; PubMed Central PMCID: PMC188991. 7707518 10.1128/jvi.69.5.2926-2931.1995 PMC188991

[38] Juuti JT, Bamford DH. Protein P7 of phage φ6 RNA polymerase complex, acquiring of RNA packaging activity by *in vitro* assembly of the purified protein onto deficient particles. J Mol Biol. 1997;266(5):891–900. Epub 1997/03/14. doi: 10.1006/jmbi.1996.0817 . 9086268 10.1006/jmbi.1996.0817

